# Using Recombinant Superoxide Dismutase to Control Oxidative Stress in the Gastrointestinal Tract of Cyclic Heat-Stressed Pigs

**DOI:** 10.3390/ani13162681

**Published:** 2023-08-21

**Authors:** Hieu Huu Le, Weicheng Zhao, John Barton Furness, Majid Shakeri, Kristy DiGiacomo, Eugeni Roura, David Renaudeau, Nicolas Kurt Gabler, Brian Joseph Leury, Frank Rowland Dunshea, Gene Wijffels, Jeremy James Cottrell

**Affiliations:** 1 Faculty of Science, The University of Melbourne, Parkville, VIC 3010, Australia; huul1@student.unimelb.edu.au (H.H.L.); weichengz@student.unimelb.edu.au (W.Z.); majid.shakeri@usda.gov (M.S.); kristyd@unimelb.edu.au (K.D.); brianjl@unimelb.edu.au (B.J.L.); fdunshea@unimelb.edu.au (F.R.D.); 2 Faculty of Animal Sciences, Vietnam National University of Agriculture, Trau Quy, Gia Lam, Hanoi 12406, Vietnam; 3 School of Animal and Comparative Biomedical Sciences, The University of Arizona, Tucson, AZ 85719, USA; 4 Department of Anatomy and Neuroscience, The University of Melbourne, Parkville, VIC 3010, Australia; j.furness@unimelb.edu.au; 5 Florey Institute of Neuroscience and Mental Health, Parkville, VIC 3010, Australia; 6 U.S. National Poultry Research Center, USDA-ARS, Athens, GA 30605, USA; 7 Centre for Nutrition and Food Sciences, Queensland Alliance for Agriculture and Food Innovation, The University of Queensland, St. Lucia, QLD 4072, Australia; e.roura@uq.edu.au; 8 PEGASE, INRAE, Agrocampus Ouest, 16 Le Clos Domaine de la Prise, 35590 Saint-Gilles, France; david.renaudeau@inrae.fr; 9 Department of Animal Science, Iowa State University, Ames, IA 50011, USA; ngabler@iastate.edu; 10 Faculty of Biological Sciences, The University of Leeds, Leeds LS2 9JT, UK; 11 CSIRO Agriculture and Food, St. Lucia, QLD 4067, Australia; gene.wijffels@csiro.au

**Keywords:** pigs, antioxidant, hot condition, inflammation, physiological responses, gut health, dietary supplementation

## Abstract

**Simple Summary:**

Global warming is increasing the impact of heat stress on pig production, particularly in tropical and subtropical areas. This impacts efficient pork production by increasing the prevalence of heat stress syndromes, partly due to oxidative stress, inflammation, and gut dysfunction. Hence, various strategies have been introduced to reduce the adverse effects of heat stress at high temperatures. Superoxide dismutase is an antioxidant enzyme that eliminates superoxide radicals and improves redox balance. Previous studies have shown that supplementation with superoxide dismutase reduces oxidative stress and inflammatory responses, but investigations into its effects on heat-stressed pigs are lacking. Therefore, this study aimed to investigate the effects of dietary supplementation with recombinant superoxide dismutase on oxidant status and inflammatory responses of growing pigs exposed to heat stress conditions.

**Abstract:**

Climate change is associated with an increased frequency and intensity of heat waves, posing a threat of heat stress to pig production. Heat stress compromises the efficiency of pig production partly due to causing oxidative stress, intestinal dysfunction, and inflammatory responses. Superoxide dismutase is an antioxidant enzyme reported to reduce oxidative stress and inflammation. Therefore, this experiment aimed to investigate whether recombinant superoxide dismutase (_r_SOD) could ameliorate oxidative stress and inflammatory responses in heat-stressed grower pigs. Sixty-four female pigs (Large White × Landrace, 27.8 ± 1.65 kg, mean ± SD) were randomly allocated to a control diet (standard grower feed, CON) or the control diet supplemented with 50 IU recombinant superoxide dismutase (_r_SOD) for 14 days. After acclimation to the diet, pigs were then housed under thermoneutral (TN, 20 °C, 35–50% relative humidity) or cyclic heat stress conditions (_C_HS, at 35 °C: 9 a.m. to 5 p.m. and 28 °C: 5 p.m. to 9 a.m., 35–50% relative humidity) for 3 days. Heat stress increased respiration rate (RR), skin and rectal temperature (RR and RT) (*p* < 0.001 for all), and reduced plasma thyroid hormone concentration (*p* < 0.001). The amount of oxidized glutathione (GSH:GSSG) was increased in the jejunum and ileum of _C_HS pigs. In the jejunum, _r_SOD also increased the amount of oxidized glutathione in both TN and _C_HS pigs, without any change in endogenous SOD activity. In the ileum, _r_SOD prevented increases in oxidized glutathione formation in the _C_HS pigs only. Taken together, this may reflect increased oxidative stress in both the jejunum and ileum in _C_HS pigs. Alternatively, _r_SOD increased the conversion of reduced to oxidized glutathione independently of _C_HS, possibly reflecting an increased overall SOD activity due to the addition of exogenous SOD. In conclusion, the use of in-feed SOD enzymes at a dose of 50 IU/kg may be a useful strategy for preventing oxidative stress in pigs.

## 1. Introduction

The effects of global warming will mean that livestock producers must contend with increased incidences of heat stress over longer periods of the year. Pigs are more susceptible to heat stress than many other animal species because they lack functional, and genetic selection for high growth rates which increases metabolic heat production [1]. Heat stress compromises production parameters in pigs and other animal species in part due to reductions in feed intake [2]. Nevertheless, pair-feeding experiments have demonstrated that the effects of heat stress on decreased production extend beyond the decrease in feed intake alone [3]. Thermoregulatory responses to heat stress include redistributing blood flow from visceral organs to the periphery, which results in reduced visceral blood flow, resulting in oxidative stress within the gastrointestinal tract (GIT) [4,5,6]. The presence of oxidative stress increases the expression of several genes that express proinflammatory cytokines as part of a localised inflammatory response [7]. In turn, the elevated inflammatory response further increases reactive oxygen species (ROS) production from immune cells [8], exacerbating the effects of heat stress on the GIT [7].

The most widely used ROS is the superoxide radical, generated by oxidative metabolism in the mitochondria and immune cells [9]. Superoxide is considered a primary free radical that promotes the formation of other ROS, such as hydrogen peroxide, hydroxyl radicals, and lipid radicals [10]. Mass removal of superoxide occurs through the paired reactions of superoxide dismutase (SOD) and either catalase or glutathione peroxidase (GP*_x_*), as well as the involvement of antioxidant vitamins such as Vitamins E and C. The addition of elevated amounts of antioxidants such as selenium (Se) and Vitamin E protects against the oxidative stress that occurs as a result of heat stress [11,12], even within the GIT [6]. However, SOD is the first line in the enzymatic antioxidant defence system, and plays a pivotal role in regulating redox balance by catalysing the dismutation of superoxide to hydrogen peroxide, which is then converted to water by catalase and GP*_x_*. Oral administration of SOD has been demonstrated to be effective in reducing intestinal disorders such as ulcerative colitis [13,14], and melon or yeast-derived SOD supplements are effective in reducing oxidative stress (OS) in the GIT during weaning stress [15]. Further, SOD has improved growth rates and reduced inflammatory markers in lipopolysaccharide-challenged piglets [16]. Therefore, the aim of this experiment was to investigate the effects of recombinant SOD on ameliorating oxidative stress in the gastrointestinal tract of heat-stressed pigs.

## 2. Materials and Methods

### 2.1. Recombinant Superoxide Dismutase Preparation

Recombinant bovine superoxide dismutase (_r_SOD) that was expressed in *Escherichia coli* (S9697, Sigma-Aldrich, St. Louis, MI, USA) was reconstituted in 10 mM potassium phosphate buffer pH 7.4 to make up a 75 UI/mL SOD solution. The enzyme solution was then aliquoted and stored at −20 °C until required.

### 2.2. Animals and Experimental Design

All experimental protocols in this study were approved by the University of Melbourne Animal Ethics Committee (Protocol no. 1714291.1). Sixty-four female growing pigs (Large White × Landrace, 27.7 ± 1.68 kg, mean live weight ± SD) were randomly assigned in a 2 × 2 factorial design (*n* = 16 per treatment) with two dietary treatments and two environmental conditions over five replicates. Pigs were randomly assigned to receive a control diet (standard grower diet) or a control diet plus 50 IU SOD/kg for 14 days. The dose and administered duration of _r_SOD were based on the study by [15]. All _r_SOD was administered on top of the feed in liquid form during each meal. The standard diet was formulated from the main ingredients of wheat and canola meal to meet the National Research Council recommendations (2012) for growing pigs. The diet contained 14.0 MJ/kg of digestible energy and 16.0% crude protein (Table 1). After 14 days of dietary acclimation, 16 pigs from each dietary treatment were moved to individual metabolism cages and housed under either TN or cyclic heat stress conditions (_C_HS). The temperature in the TN condition was set at a constant 20 °C. The temperature in the _C_HS condition was set at a 35 °C from 9 a.m. to 5 p.m. and 28 °C from 5 p.m. to 9 a.m. on the following day for 3 days (*n* = 32 for each temperature condition). Relative humidity was set between 30 and 50% for both temperature groups. To avoid differences in voluntary feed intake resulting from _C_HS, throughout the experiment, the pigs were fed a restricted diet at 2.5 times of energy requirement for maintenance (approximately 80% of ad libitum intake), and feed allowance was adjusted weekly based on body weight change. Water was supplied ad libitum via nipple drinkers. No prophylactic treatment was administered throughout the experiment.

### 2.3. Physiological Observations

Physiological parameters of each pig were monitored at 0900, 1100, 1300, 1500, and 1700 h during the first 2 days of heat exposure. The respiration rate (RR, breaths/min.) was measured by counting flank movements within 20 s using a stopwatch (Digital timer PTR388, Labtek Pty Ltd., Brendale, QLD, Australia), then expressed as breaths per minute. Rectal temperature (RT) was measured by inserting the probe of a digital rectal thermometer (Surgipack, Medtronic Australasia Pty Ltd., Richmond, VIC, Australia) approximately 2 cm into the rectum. Skin temperature was measured using a non-contact digital InfraRed Thermometer (QM7721, Non-contact IR thermometer, Digitech, New York, NY, USA) targeting the skin at the shoulder until a stable temperature was recorded.

### 2.4. Blood Collection and Measurement

At the end of the experiment (on day 3 of the _C_HS challenge), pigs were sedated with an intramuscular injection of ketamine (20 mg/kg; Troy Laboratories Pty Ltd., Glendenning, NSW, Australia) and xylazine (3 mg/kg; Troy Laboratories Pty LTD, NSW, Australia). After deep anaesthesia was confirmed by the absence of normal reflexes, including eye and pinch tests, a fresh venous blood sample (1 mL, *n* = 16 per treatment) was withdrawn from the ear vein and immediately analysed for blood oximetry and biochemistry (EPOC^®^; Alere, Waltham, MA, USA) for determination of blood gas parameters and chemistry. After that, a 5 mL blood sample (*n* = 16 per treatment) was obtained from the jugular vein using a 10 mL anticoagulant vacutainer tube (Lithium heparin-coated, BD vacutainer^®^, BD Australia, North Ryde, NSW, Australia). The vacutainer tubes were then centrifuged for 10 min. (2000× *g*, 4 °C) and plasma was collected and then stored at −20 °C until analysis. The estimated glomerular filtration rate (_e_GFR) was estimated from blood creatinine and body weight using an equation given by Gasthuys and co-workers [17]:

(1) $$eGFR=\frac{1.879\times {BW}^{1.092}}{P_{Cr}^{0.600}}$$

where:

- _e_GFR is the estimated glomerular filtration rate (mL/min);
- BW is body weight (kg);
- P_Cr_ is plasma creatinine concentration (mg/dL).

### 2.5. Plasma Hormones and Metabolite Measurement

The plasma cortisol concentration was measured using a radioimmunoassay kit (ImmuChem^TM^ Coated Tube Cortisol 125I RIA kit, MP Biomedicals, Diagnostics Division, Orangeburg, NY, USA) according to the manufacturer’s instructions. The minimum detectable dose of cortisol in the assay was 1.7 ng/mL, and the mean intra-assay coefficients of variation (CVs) were 2.95%.

For thyroid hormones, triiodothyronine (free T_3_) and free thyroxine (free T_4_) were measured using radioimmunoassay kits (Cat. 06B258710, 06B257215, MP Biomedicals, Orangeburg, NY, USA) according to the manufacturer’s instructions. The detection limit and intra-assays CV of T_3_ were 0.06 pg/dL and 5.12%, respectively, while values for T_4_ were 0.45 pg/dL and 5.12%.

Plasma concentration of glucose (Cat no. TR15221), urea nitrogen (Cat no. TR12421), and triglycerides (Cat no. 981786) were measured using reagents supplied from Thermo Fisher Scientific (Waltham, MA, USA) as per the manufacturer’s protocol. The inter- and intra-assay CVs of 3.87% and 3.22% for glucose, 3.57% and 3.23% for PUN, and 3.8% and 2.1% for triglycerides were found. Plasma creatinine concentration was measured using a kit (Cat no. CR510, Randox, County Antrim, BT29 4QY, UK) as per the manufacturer’s instructions, and the inter- and intra-CV were 0.92% and 1.93%, respectively.

### 2.6. Antioxidant Assays

Total antioxidant capacity of plasma (TAC) was quantified as per the manufacturer’s protocol (Cat no. 709001, Cayman Chemical, Ann Arbor, MI, USA). The results of TAC were expressed as Trolox millimolar equivalent, where the average inter- and intra-assay CVs were 2.04 and 1.47%, respectively. Plasma activities of superoxide dismutase (SOD) and glutathione peroxidase (GP*_x_*) were measured using commercial kits (Cat no. 706002 and 703102, Cayman Chemical, Ann Arbor, MI, USA) as per the manufacturer’s instructions. The results of TAC were expressed as Trolox-equivalent antioxidant capacity, with the average inter- and intra-assay CV being 2.04 and 1.47%, respectively. One unit of SOD is defined as the amount of enzyme needed to exhibit 50% dismutation of superoxide radical. Meanwhile, for GP*_x_*, it was defined as the amount of enzyme that will cause the oxidation of 1.0 nmol of NADPH to NADP^+^ per minute at 25 °C.

### 2.7. Heat Shock Protein, Cytokines and Metabolic Hormones

Plasma concentrations of heat shock protein 27 (Cat DY1580) and heat shock protein 70 (Cat DY1663–05) (In Vitro Technologies, Noble Park, VIC, Australia) were used as parameters for heat stress markers. Enzyme-linked immunosorbent assays were performed to measure the following inflammatory cytokines using kits as per the manufacturer’s instructions (R&D Systems, Minneapolis, MI, USA): interleukin 1β (Cat DY681), interleukin 6 (Cat DY686), interleukin 10 (Cat DY693B), and tumour necrosis factor-α (Cat DY690B) (In Vitro Technologies, Noble Park, VIC, Australia). Metabolic hormones, adiponectin and leptin, were measured as described in [18].

### 2.8. Urine Analysis

Immediately after pigs were euthanised via intracardiac barbiturate overdose (Sodium pentobarbitone; 162.5 mg/kg liveweight; Lethabarb; Virbac, NSW, Australia) and the abdomen was opened along the mid-line abdominal wall, urine samples (*n* = 16 per treatment) were collected directly from the bladder with a syringe. Urine pH was measured immediately using a freshly calibrated pH meter (Eutech pH 5+, Thermo Fisher Scientific, Waltham, MA, USA). Osmolality was determined using an osmometer (Advanced Micro Osmometer 3300, Advanced Instruments, Norwood, MA, USA) and expressed as mOsm/kg H_2_O. Creatinine and bilirubin were quantified using kits as per the manufacturer’s instructions (Creatinine Urinary Colorimetric Assay Kit, Cayman Chemical, Ann Arbor, MI, USA and Cat no. 981897, Thermo Fisher Scientific, Waltham, MA, USA, respectively). The inter- and intra-assay CVs were 3.1% and 1.0% for creatinine and 8.4% and 2.7% for bilirubin.

### 2.9. Sample Collection and Intestinal Integrity Measurement

Sections of the proximal jejunum, distal ileum, and colon were collected immediately after euthanasia (*n* = 16 per treatment for each). Fresh intestinal samples were rinsed and placed in a chilled intravenous infusion of 0.9% sodium chloride (Baxter, Old Toonggabie, NSW, Australia). The sections were then transferred to Krebs solution (11.1 mM glucose, 118 mM NaCl, 4.8 mM KCl, 1.0 mM NaH_2_PO_4_, 1.2 mM MgSO_4_, 25 mM NaHCO_3_, 2.5 mM CaCl_2_, pH 7.4). After removal of the muscularis propria, the mucosal layer was mounted on a round slider (area of 0.71 cm^2^) and then placed in a two-part Ussing chamber (EasyMount Diffusion Chambers, Physiologic Instruments, San Diego, CA, USA). Measurement of intestinal transepithelial resistance (TER) and transport of fluorescein isothiocyanate dextran (4 kD, FD4) was performed according to the methods described by Cottrell and co-workers [19]. The TER and FD4 permeability values were expressed as Ω.cm^2^ and apparent permeability (P_app_, 10^−4^ cm/sec). Aliquots of each tissue section were placed in a 5 mL polypropylene screw cap tube (SARSTEDT AG & Co. KG, Nümbrecht, Germany) and immediately frozen in liquid nitrogen The tubes were then stored at −80 °C until analysis.

### 2.10. Intestinal Oxidative Stress Biomarkers Measurement

Frozen specimens from jejunum and ileum (*n* = 16 per treatment for each organ) were pulverised in a mill pre-chilled with liquid nitrogen (Cellcrusher, Cork, Ireland). To measure SOD activity, a powdered frozen sample (0.1 g) was homogenised in 1 mL of 20 mM HEPES cold buffer (1 mM EGTA, 210 mM mannitol, and 70 mM sucrose, pH 7.2), then was centrifuged at 1500× *g* for 5 min at 4 °C to collect the supernatant for the SOD activity assay as per Section 2.6. For GP*_x_* activity measurement, pulverised samples (0.1 g) were homogenised in 1 mL Tris-HCl buffer (50 mM Tris-HCl, 5 mM EDTA, 1 mM DTT, and pH 7.5). The supernatant was obtained after the homogenate was centrifuged at 10,000× *g* for 15 min at 4 °C, and GP*_x_* activity was measured as described in Section 2.6. The concentration of reduced glutathione (GSH) and oxidised glutathione (GSSG) concentration were measured using a glutathione assay kit (Cat no. 703002, Cayman, Ann Arbor, MI, USA) as per the manufacturer’s instructions. Briefly, powdered samples (0.1 g) were homogenised in 1 mL of cold buffer (50 mM MES, pH 6–7, containing 1 mM EDTA). Homogenates were centrifuged at 10,000× *g* for 15 min at 4 °C to collect supernatants. The supernatants were deproteinized before performing the assays as follows: an equal volume of metaphosphoric acid solution (5 g/50 mL water) was added to the supernatant and mixed by vortexing. The mixture was allowed to stand at room temperature for five minutes before being centrifuged at 2000× *g* for 5 min at 20 °C to obtain the supernatant. Triethanolamine solution (4 M, 50 µL) was added to the supernatant (1 mL), then vortexed, and the sample was ready for the GSH assay. For the GSSH assay, after being mixed with 4 M triethanolamine solution, 1 mL of the sample was treated with 10 µL of the 1 M 2-vinylpyridine solution. The mixture was vortexed and incubated at room temperature for 60 min before assaying. The activity of SOD and GP*_x_* were normalized to the protein concentration, which was measured using the Pierce^TM^ Coomassie protein assay kit (Cat no. 23200, Thermo Fisher, Waltham, MI, USA).

### 2.11. Statistical Analysis

All data were verified through a normal distribution and analysed using a general analysis of variance (Genstat v.18th VSN International, Hemel Hempstead, UK). For respiration rate, rectal temperature, and skin temperature, data were analysed for the main effect of temperature (TN vs. _C_HS), diet (CON vs. _r_SOD), time of the day, and the interactions, and then the results were presented graphically. For blood gas parameters, plasma and urine metabolites, TER, FD4 permeability, oxidative stress biomarker, heat shock protein, and inflammatory markers, the effects of temperature (TN vs. _C_HS) and diet (CON vs. _r_SOD) were analysed using individual pigs as experimental units and experimental replication as a blocking factor. If data were skewed, a log_10_ transformation was conducted to reduce the heterogeneity of variances before ANOVA analysis. In the text, data were presented as the adjusted means and standard error of the difference (SED) for the pooled main effects of temperature (T) and diet (D), while in figures and tables, data were expressed as adjusted means and SED for the full interactions (temp × diet × time). Differences were considered significant if the *p*-value ≤ 0.05, while a trend was considered if the *p*-value ≤ 0.10. Fisher’s least significant difference test was used post hoc to compare multiple means, with alphabetical superscripts used to identify different groups at *p* ≤ 0.05.

## 3. Results

### 3.1. Physiological Responses

**Figure 1:**
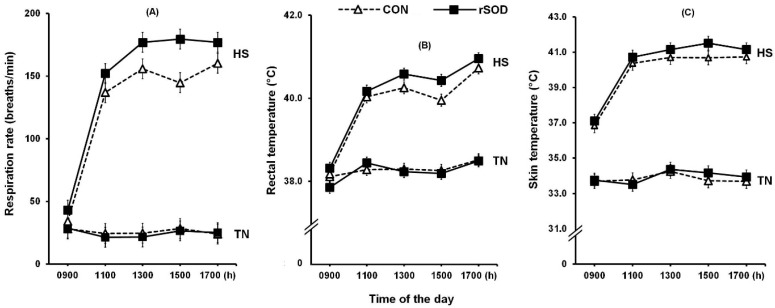
(**A**) Respiration rate, (**B**) rectal temperature and (**C**) skin temperature of pigs fed a control diet (CON) or the control diet plus 50 IU _r_SOD/kg (SOD) under either thermoneutral (TN) or cyclic heat stress (_C_HS) conditions. Each panel represents the adjusted means with pooled standard error of means for temp × diet × time interaction for 2 days of _C_HS challenge. Panel (**A**) indicated the respiration rate was significantly increased by _C_HS (*p* < 0.001), and time of the day (*p* < 0.001), and was higher with _r_SOD overall (*p* = 0.018) and under _C_HS (temp × diet, *p* < 0.001). Panel (**B**) showed that the rectal temperature was significantly increased by _C_HS (*p* < 0.001), time of the day (*p* < 0.001) and tended to be increased by _r_SOD (*p* = 0.077), and increased with _r_SOD under _C_HS (temp × diet, *p* < 0.001). Panel (**C**) showed the skin temperature was increased by _C_HS (*p* < 0.001), time of the day (*p* < 0.001) and was higher with _r_SOD overall (*p* = 0.048). There was no interaction of temp × diet on ST, and each group represents *n* = 16 pigs.

The pig’s RR was increased by _C_HS (25 vs. 146 breaths/min for TN vs. _C_HS, *p* < 0.001). There was an overall effect of time and an interaction between temperature and time such that RR increased overall (33.4^a^, 88.2^b^, 100^c^, 97^c^ and 102^c^ breaths/min at 0900, 1100, 1300, 1500 and 1700 h, respectively; *p* < 0.001), but this was with the _C_HS pigs only, which increased from 39 to 185 breaths/min between 0900 and 1700 h, while in TN pigs, it remained stable at around 25 breaths/min during the same period (temp × time interaction *p* < 0.001, Figure 1A). Dietary supplementation with _r_SOD increased overall RR compared to CON (80 vs. 88 breaths/min, *p* = 0.003), which appeared to be due to elevations in the _C_HS pigs (136 vs. 156 breaths/min, *p* < 0.001) but not under TN conditions (Figure 1A). There were no interactions between diet and time or temperature, diet, and time.

Rectal temperature was increased by _C_HS (38.3 vs. 39.9 °C, *p* < 0.001) and overall time of the day (38.1^a^, 39.2^b^, 39.3^b^, 39.2^b^ and 39.7^c^ °C at 900, 1100, 1300, 1500 and 1700 h; *p* < 0.001; Figure 1B). This was due to increases in the _C_HS pigs, which increased from 38.3 °C to 40.8 °C between 0900 and 1700 h, while in TN pigs, there was a small increase from 38.0 °C to 38.5 °C over the same period (temp × time interaction *p* < 0.001). Rectal temperature tended to be increased with _r_SOD (39.0 vs. 39.1 °C, *p* = 0.077). There was an interactive effect between _C_HS and diet (*p* < 0.001), such that feeding _r_SOD increased the RT of _C_HS pigs only (39.8 vs. 40.1 °C). There were no interactions between diet and time or temperature, diet, and time (Figure 1B).

Skin temperature was increased by _C_HS (33.9 vs. 40.1 °C, *p* < 0.001) and by time of the day (35.4^a^, 37.1^b^, 37.6^c^, 37.5b^c^ and 37.3^bc^ °C at 0900, 1100, 1300, 1500 and 1700 h, respectively; *p* < 0.001; Figure 1C). This was due to increases in the _C_HS pigs, which increased from 37.0 °C to 40.9 °C between 0900 and 1700 h, while TN pigs exhibited a small increase from 33.7 °C to 33.8 °C over the same period (temp × time interaction *p* < 0.001, Figure 1C). Skin temperature was elevated by _r_SOD (36.9 vs. 37.1 °C, *p* = 0.048). There were no interactions between diet and time or temperature, diet, and time on ST.

### 3.2. Blood Biochemistry

There were no main or interactive effects of _C_HS and _r_SOD on blood pH (Table 2). Blood partial pressure of CO_2_ (49.4 vs. 46.2 mmHg, *p* < 0.001) and total CO_2_ (35.0 vs. 32.4 *p* < 0.001) were decreased by _C_HS but they were unchanged by _r_SOD supplementation. Blood bicarbonate (HCO_3_) was reduced by _C_HS (33.8 vs. 31.0 mM, *p* < 0.001), but was not affected by diet. The blood’s partial pressure of O_2_ (65.0 vs. 52.1 mmHg, *p* < 0.001) and O_2_ saturation (91.8 vs. 86.5%, *p* < 0.001) were decreased by _C_HS, whereas there was no main effect of diet. Blood base excess was reduced by _C_HS (8.37 vs. 5.98 mM, *p* < 0.001), but it was not influenced by diet. Blood haematocrit (34.3 vs. 31.0%, *p* < 0.001) and blood haemoglobin (11.7 vs. 10.5 g/dL, *p* < 0.001) were decreased by _C_HS while there were no significant effects of diet or interaction. Blood potassium concentrations were increased by _C_HS (4.02 vs. 4.24 mM, *p* = 0.001), whereas they were not affected by diet. In contrast, blood sodium concentrations were decreased by _C_HS (144 vs. 143 mM, *p* = 0.037) and _r_SOD (144 vs. 143 mM, *p* = 0.059). While the blood anion gap was not affected by _C_HS, it tended to be decreased by _r_SOD (13.4 vs. 14.3 mM, *p* = 0.080). Blood lactate (1.71 vs. 1.16 mM, *p* = 0.002) and creatinine (2.07 vs. 1.74 mg/dL, *p* < 0.001) concentrations were decreased by _C_HS, whereas there were no main or interactive effects of diet. Neither _C_HS nor diet influenced blood concentrations of chloride, calcium and glucose (Table 2).

### 3.3. Plasma Biochemistry

The results of plasma biochemistry were presented in Table 3. With the exception of triglycerides, no main or interactive effects of diet were observed; so, the effects of _C_HS will be summarised first. Parameters that were reduced by _C_HS include plasma T_3_ (0.883 vs. 0.493 pg/mL, *p* < 0.001), T_4_ (18.7 vs. 13.1 pg/mL, *p* < 0.001), the T_3_-to-T_4_ ratio (0.047 vs. 0.037, *p* = 0.001) and _e_GFR (74.4 vs. 67.7 mL/min/kg, *p* = 0.020, Table 3). Parameters that were increased by _C_HS include plasma glucose (6.92 vs. 7.20 mM, *p* = 0.008) and creatinine was increased by _C_HS (1.36 vs. 1.59 mg/dL, *p* = 0.004, Table 3). There was an indication of an interaction between temperature and diet (*p* = 0.062) such that pigs fed _r_SOD exhibited a lower triglyceride than those pigs fed CON, but this difference appears during _C_HS only. No effects or interactions of temperature and diet were observed on plasma protein, plasma urea nitrogen or cortisol. Plasma SOD activity was decreased by _C_HS (3.00 vs. 2.46 IU/mL, *p* = 0.032, Table 3), and no main or interactive effects of diet were observed. Total antioxidant capacity and glutathione peroxidase activity (GP_x_) were not influenced by _C_HS or diet.

Overall _C_HS increased IL-1β (0.39 vs. 0.82 ng/mL, *p* = 0.013) and reduced TNF-α (4.40 vs. 2.54 ng/mL, *p* = 0.043, Table 4). Alternatively, _r_SOD reduced IL-1β (0.84 vs. 0.42 ng/mL, *p* = 0.015) and IL-10 (0.58 vs. 0.133 ng/mL). Interactions between temperature and diet were observed such that _r_SOD increased adiponectin and HSP27 under TN but not _C_HS conditions (Table 4). No effects of diet on TN IL-10 concentrations were observed, but there was an increase in CON but not _r_SOD pigs under _C_HS conditions. No effects of interactions of temperature or diet were observed on leptin, IL-6, and HSP70.

### 3.4. Urinary Biochemistry

Cyclic heat stress reduced urinary pH (6.10 vs. 5.82, *p* = 0.009) but increased urine osmolality (294 vs. 468 mOs/kg H_2_O, *p* = 0.009) and bilirubin (17.8 vs. 28.6 µM, *p* = 0.023, Table 5). There was a tendency for osmolarity (313 vs. 441 mOs/kg H_2_O, *p* = 0.074) and bilirubin (18.1 vs. 27.6 µM, *p* = 0.070) to be increased in _r_SOD-supplemented pigs, but no interaction with temperature was observed. Urinary creatinine was increased by _C_HS (94.2 vs. 156 mg/dL, *p* = 0.016), with no dietary or interactive effects observed (Table 5).

### 3.5. Intestinal Antioxidant Biomarkers

The effects of _C_HS on GP*_x_* differed by intestinal segment, being lower in the jejunum (75.2 vs. 66.0 IU/mg protein, *p* = 0.008) but greater in the ileum (65.4 vs. 74.0 IU/mg protein, *p* = 0.027) (Table 6). The activity of SOD was not influenced by _C_HS. The jejunum GSH:GSSG ratio was reduced by _C_HS (2.14 vs. 2.00, *p* = 0.013) and _r_SOD (2.14 vs. 2.01, *p* = 0.049), but no interaction with _C_HS was observed. Dietary _r_SOD did not influence GP*_x_* activity in either the jejunum or the ileum. While there were no main effects of _C_HS or diet on the ileal GSH:GSSG ratio, there was a trend for an interaction (*p* = 0.069) such that during _C_HS, the GSG:GSSG ratio was decreased to a greater extent in pigs fed the CON diet than those on the _r_SOD diet.

### 3.6. Intestinal Transepithelial Resistance and Permeability

No effects of temperature or diet were observed on transepithelial resistance or FD4 permeability in the small intestine and colon (Table 7).

## 4. Discussion

Heat stress is known to induce oxidative stress in the GIT of the pig. The objective of this experiment was to determine if the addition of SOD in a recombinant form with feed could ameliorate the impact of _C_HS in the pig. The model used in this experiment elicited a robust heat stress response. Furthermore, parameters of OS such as oxidised glutathione were elevated in the jejunum and ileum of _C_HS pigs, suggesting that the model induced small-intestinal oxidative stress. However, it is important to note that the disruption of the mucosa associated with OS [6] was not observed in this study. This increase in parameters of OS was in part ameliorated by the addition of dietary _r_SOD in the ileum but not the jejunum, indicating that _r_SOD had protective effects against OS in the GIT of the _C_HS pig. However, there were also cautionary results, with increases in the physiological indices of heat stress, such as respiration rate, and rectal and skin temperature in _r_SOD-supplemented pigs, indicating the potential for _r_SOD to exacerbate some indices of heat stress.

A study of how heat stress alters the proteome in pigs showed that antioxidant pathways, specifically GP*_x_*, were among the most affected pathways [20]. Previous results from our laboratory have supported this observation, with reductions in GP*_x_* activity and increases in oxidised glutathione observed in pigs [6]. In this experiment, the overall effect of _C_HS was to increase GIT OS, as evidenced by elevated glutathione oxidation in both the jejunum and ileum. In mixed venous plasma, TAC was used as a marker of oxidative stress, with no differences between TN and _C_HS pigs. The apparent difference between markers of OS in the small intestine and venous plasma may be an artefact of the different measurement procedures. However, the more likely scenario is that this result reflects localised oxidative stress in the small intestine. If the heat treatment resulted in a larger insult, the OS markers may be evident in plasma. However, unlike previous studies conducted by our laboratory [6,19,21] and others [20,22], the extent of heat stress on the pigs did not result in a disruption of GIT barrier function, which would then contribute to an increase in plasma-borne markers.

In the jejunum rSOD had an overall increase in the formation of oxidised glutathione, as indicated by a reduced GSH:GSSG. The increased glutathione oxidation is consistent with increased SOD activity. As there were not any significant increases in jejunal SOD activity, it is possible that the increase in glutathione oxidation may be due to the exogenous rSOD supplied within the diets rather than an increased SOD expression. In both the jejunum and ileum, _C_HS did not influence tissue SOD activity, but different outcomes in glutathione oxidation and GP*_x_* activity were observed. In the jejunum, there was an increase in glutathione oxidation and a decrease in GP*_x_* overall activity with _C_HS, consistent with oxidative stress. In the ileum, there was an increase in GP_x_ activity with _C_HS, but glutathione oxidation only increased in the _C_HS control group. When put together, it appears that this model of _C_HS induced oxidative stress in both the jejunum and ileum. The effects of _r_SOD appear to be an increase in glutathione oxidation in the jejunum and a prevention of oxidative stress induced by _C_HS in the ileum. The increase in jejunum glutathione oxidation requires further investigation, as it may reflect oxidative stress, but could also reflect increased activity of antioxidant pathways.

Elsewhere, recombinant-derived SOD has been used to prevent ulcerative colitis in experimental models [23]. However, the same experiment demonstrated that conjugation with soy lecithin improved the biological activity and forms of SOD derived from melon pulp extracts have been shown to improve layer hen performance and egg quality, and to ameliorate post-weaning mucosal atrophy [15,24]. Recombinant *SodA* (Superoxide dismutase)-expressing lactobacillus demonstrated protective effects in murine colitis models [25]. Therefore, there is compelling evidence for the beneficial effects of exogenous SOD within the gastrointestinal tract. The SOD used in this experiment was not a protected form and the effects on glutathione oxidation were only observed in the jejunum. This likely reflects the digestion of _r_SOD within the GIT. Nevertheless, an apparent beneficial effect of _r_SOD was observed with an apparent reduction in oxidative stress, which presumably was due to upstream effects. This experiment represents the first indication that exogenous SOD affects glutathione oxidation and prevents oxidative stress in the GIT of pigs using a _C_HS model. Furthermore, it is possible this outcome may be improved using SOD formulations within a protective matrix that extend the half-life of exogenous SOD activity within the gastrointestinal tract. Overall, there was no effect of either _C_HS or _r_SOD on parameters of GIT integrity (P_app_, TER); so, no conclusions are able to be made as to whether the apparent reduction in oxidative stress provided an improvement in gut health.

Other effects of _r_SOD were an apparent deterioration of physiological parameters of _C_HS. This was not accompanied by changes in thyroid hormones or blood gas analysis that would be expected to be accompanied with an increase in _C_HS. Furthermore, _r_SOD resulted in an overall reduction in IL-1β and prevented increases in IL-10 in the _C_HS pigs. This could indicate alterations to the inflammatory response, but since IL-1β and IL-10 are associated with fever formation, this result may indicate an adaptative response. The authors have considered that this may be in part due to the recombinant SOD being produced from an *E. coli* expression system and that there may have been endotoxin contamination [26]. This was ruled out because there was a lack of a proinflammatory response with the _r_SOD diet and that it appeared to have an anti-inflammatory response in the _C_HS pigs. Regardless, it cannot be concluded that the increase in _C_HS indicated by the physiological parameters indicated a worsening of heat stress as it was not accompanied by a deterioration in tissue or plasma biomarkers.

Heat stress is known to reduce lipid oxidation and promote fat deposition in growing pigs [27,28]. In this study, _C_HS increased plasma triglyceride concentration by 22%, suggesting increased triglyceride synthesis, possibly due to the glyceroneogenesis in liver and adipose tissue during heat stress conditions [29,30]. Interestingly, _r_SOD supplementation reduced plasma triglycerides by 15% during _C_HS, indicating protection against hypertriglyceridemia. This effect of SOD on triglyceride agreed with research in mice and humans. Mice fed a high-fat diet and treated with nano-sized SOD had lower serum and hepatic triglyceride concentrations than their contemporaries [31]. The nano-sized SOD diet decreased liver levels of SREBP-1c, a transcription factor that supports lipogenesis, and increased anti-inflammation in ethanol-treated mice [32]. As elevated plasma triglycerides are commonly associated with insulin resistance [33,34], the lowered plasma triglyceride concentrations suggest that _r_SOD supplementation could protect against hypertriglyceridemia and insulin resistance in heat-stressed pigs.

The effects of heat stress on renal function in livestock is a topic that requires further investigation. This study showed that _C_HS decreased eGFR and increased plasma creatinine concentration, indicating reduced renal creatine clearance and possibly reduced renal function. Biomarkers of renal injury have been observed in athletes competing under hot conditions [35,36] and people with renal disease are overrepresented in hospital admissions during heat waves [37]. There have been intensive research efforts to characterise the effects of heat stress on the GIT and studies of regional changes in blood flow found reduced renal blood flow in sheep and rats [38,39]. Reduced blood flow is postulated to be a causative factor towards the loss of mucosal integrity following heat stress and reductions in renal blood flow may also compromise renal function during heat stress. Studies using Evans blue as an exogenous marker of vascular permeability demonstrated increased extravasation in the heat-stressed broiler [40], and preliminary findings in pigs support this observation [41]. Previously, we have observed that heat stress increases urinary acidity [19], a result confirmed by the current experiment. There was also an increase in urinary creatinine, but as this determined from a single urine sample taken at the point of euthanasia, it is difficult to extrapolate this to renal function. For example, the concomitant increase in urinary osmolarity may reflect concentration of the urine, and the increase in creatinine concentrations may instead reflect hypovolemia and dehydration due to the increased environmental temperature and not a change in creatinine clearance.

## 5. Conclusions

The key findings of this research were that oxidized glutathione concentrations were increased in the GIT by both _C_HS and _r_SOD. The authors believe that the reasons for this differ, with increased in glutathione oxidation during _C_HS a product of increased free radicals and oxidative stress. The addition of _r_SOD increased glutathione oxidation irrespective of _C_HS, indicating an alternative mechanism. We propose that this reflected an increase in overall SOD activity, resulting in an increased formation of oxidized glutathione. Further protective effects included a reduction in _C_HS-mediated lipolysis and reductions in cytokines IL-1β and IL-10, which due to their role in fever formulation, may indicate an adaptive thermoregulatory role. However, there was an apparent decline in the physiological indices of _C_HS with _r_SOD, but this was not associated with a corresponding decline in biochemical parameters. Collectively, these results indicate that the administration of 50 IU/kg of exogenous _r_SOD can influence oxidative stress in the GIT of pigs and may be a useful countermeasure against HS.

## Figures and Tables

**Table 1:** Ingredient and nutritional compositions of the control diet (as fresh feed basis).

Items	Amount
Ingredient	%
Wheat	71.1
Barley	10.0
Canola meal 37%	10.0
Soybean meal 46.5%	3.30
Meat meal 60%	1.70
Canola oil	1.00
Limestone	1.00
Dicalphos Bin Add	0.70
Lysine-HCl	0.520
DL-Methionine	0.060
Vitamin E 50% premix	0.080
Threonine micro	0.20
Salt Bin micro	0.20
Premix	0.10
Calculated values	
Digestible energy (MJ/kg)	14.0
Crude protein (%)	16.0
Lysine (%)	1.03
Available lysine (%)	0.91
Methionine (%)	0.313
Calcium (%)	0.778
Total phosphorous (%)	0.580

**Table 2:** Effect of recombinant superoxide dismutase (_r_SOD) supplementation on blood gases and acid–base balance of growing pigs under thermoneutral (TN) or cyclic heat stress (_C_HS) conditions.

Variables	TN		_C_HS		SED ^1^	*p*-Value ^2^	
	CON	_r_SOD	CON	_r_SOD		T	D
pH	7.45	7.44	7.43	7.44	0.010	0.26	0.93
Haematocrit, %	34.4	34.2	30.6	31.5	1.07	<0.001	0.74
Haemoglobin, g/dL	11.7	11.6	10.4	10.7	0.35	<0.001	0.81
Oximetry							
pO_2_, mmHg	65.1	64.0	52.1	52.1	3.75	<0.001	0.72
O_2_ saturation, %	92.6	91.2	87.1	86.0	1.82	<0.001	0.34
pCO_2_, mmHg	48.7	50.1	46.7	45.6	1.48	0.004	0.93
Total CO_2_, mM	35.0	35.0	32.7	32.1	0.94	<0.001	0.70
Ions							
HCO_3_, mM	33.5	34.1	31.3	30.7	0.83	<0.001	0.92
Potassium, mM	4.03	4.02	4.19	4.31	0.088	0.001	0.40
Sodium, mM	144	145	143	144	0.7	0.037	0.059
Chloride, mM	100	101	100	102	1.4	0.34	0.11
Calcium, mM	1.41	1.39	1.38	1.36	0.027	0.15	0.29
Base excess, mM	8.25	8.49	6.21	5.74	0.764	<0.001	0.94
Anion gap, mM	13.2	13.9	13.6	14.6	0.66	0.32	0.080
Metabolites							
Glucose, mM	6.58	6.47	6.51	6.85	0.352	0.58	0.63
Lactate, mM	1.57	1.84	1.13	1.20	0.237	0.002	0.48
Creatinine, mg/dL	2.05	2.09	1.74	1.73	0.131	<0.001	0.82

^1^ SED: standard error of the difference for the interaction between temperature and diet. ^2^ T: temperature; D: diet. T × D interaction were not significant for all parameters. *n* = 16 pigs per group.

**Table 3:** Effect of recombinant superoxide dismutase (_r_SOD) supplementation on plasma metabolites of growing pigs under thermoneutral (TN) or cyclic heat stress (_C_HS) conditions.

Variables ^1^	TN		_C_HS		SED ^2^	*p*-Value ^3^	
	CON	_r_SOD	CON	_r_SOD		T	D
Protein, mg/mL	56.9	57.6	55.3	58.1	3.33	0.83	0.61
Glucose, mg/dL	6.87	6.96	7.11	7.29	0.139	0.008	0.17
Creatinine, mg/dL	1.36	1.36	1.58	1.60	0.112	0.004	0.78
eGFR, mL/min/kg	72.4	76.4	68.7	66.7	3.88	0.020	0.72
PUN, mM	17.9	17.9	18.0	17.9	0.07	0.40	0.12
PUN to Creatinine	38.8	42.5	43.6	33.8	7.64	0.68	0.59
Triglycerides ^4^, µM	0.38 ^a^	0.45 ^ab^	0.53 ^b^	0.45 ^a^	0.060	0.068	0.92
Cortisol, ug/mL	55.0	61.9	55.2	58.7	6.07	0.70	0.37
T_3_, pg/mL	0.853	0.911	0.549	0.434	0.0792	<0.001	0.50
T_4_, pg/mL	18.0	19.4	13.4	12.7	1.39	<0.001	0.94
T_3_/T_4_	0.045	0.048	0.039	0.036	0.0041	0.003	0.95
TAC, mmol Trolox	3.23	3.63	3.15	3.04	0.420	0.33	0.31
GP*_x_*, IU/mL	310	255	294	298	44.2	0.50	0.66
SOD, IU/mL	3.27	2.72	2.36	2.57	0.352	0.032	0.45

^1^ eGFR: estimated glomerular filtration rate; PUN: plasma urea nitrogen; T_3_: triiodothyronine; T_4_: thyroxine; TAC: total antioxidant capacity; GP*_x_*: glutathione peroxidase; SOD: superoxide dismutase. ^2^ SED: standard error of the difference for the interaction between temperature and diet. ^3^ T: temperature; D: diet. T × D interactions were not significant for all parameters except where indicated. ^4^ T × D interaction (^a,b^ *p*-value = 0.062). *n* = 16 pigs per group.

**Table 4:** Effect of recombinant superoxide dismutase (_r_SOD) supplementation on plasma biomarkers of heat stress and inflammation in growing pigs housed under thermoneutral (TN) or cyclic heat stress (_C_HS) conditions.

Variables ^1^	TN		_C_HS		SED ^2^	*p*-Value ^3^	
	CON	_r_SOD	CON	_r_SOD		T	D
Adiponectin ^4^, µg/mL	5.75 ^a^	7.27 ^b^	6.97 ^ab^	6.14 ^ab^	0.792	0.85	0.45
Leptin, ng/mL	0.80	0.69	0.73	0.69	0.106	0.71	0.31
IL-1β, ng/mL	0.46	0.33	1.16	0.51	0.221	0.013	0.015
IL-6, ng/mL	40.0	40.1	39.5	35.7	3.88	0.56	0.96
IL-10 ^5^, ng/mL	0.134 ^a^	0.150 ^ab^	0.180 ^a^	0.117 ^b^	0.0178	0.55	0.037
TNF-α ^6^, ng/mL	0.647	0.638	0.406	0.402	0.1548	0.043	0.82
	(4.45	(4.35)	(2.55)	(2.52)			
HSP27 ^7^, ng/mL	54.4 ^a^	88.4 ^b^	85.8 ^b^	71.1 ^ab^	12.99	0.41	0.20
HSP70, ng/mL	3.16	3.21	3.38	2.94	0.651	0.99	0.87

^1^ IL: interleukin; TNF-α: tumour necrosis factor-alpha; HSP: heat shock protein. ^2^ SED: standard error of the difference for the interaction between temperature and diet. ^3^ T: temperature; D: diet, T × D interactions were not significant for all parameters except where indicated. *n* = 16 pigs per group. ^4^ T × D interaction (^a,b^ *p*-Value = 0.052). ^5^ T × D interaction (^a,b^ *p*-Value = 0.004).^6^ log-transformed data and the back-transformed values were presented in parentheses. ^7^ T × D interaction (^a,b^ *p*-Value = 0.011).

**Table 5:** Effect of recombinant superoxide dismutase (_r_SOD) supplementation on urinary metabolites of growing pigs under thermoneutral (TN) or cyclic heat stress (_C_HS) conditions.

Variables ^1^	TN		_C_HS		SED ^2^	*p*-Value ^3^	
	CON	_r_SOD	CON	_r_SOD		T	D
pH	6.13	6.08	5.80	5.85	0.141	0.009	0.87
OSM, mOs/kg H_2_O	250	332	377	563	97.3	0.011	0.074
Creatinine, mg/dL	88	97	131	180	38.1	0.023	0.35
Bilirubin, µmol/L	15.8	19.6	20.5	36.5	7.51	0.041	0.070

^1^ OSM: osmolality. ^2^ SED: standard error of the difference for the interaction between temperature and diet. ^3^ T: temperature; D: diet, T × D interactions were not significant for all parameters. *n* = 16 pigs per group.

**Table 6:** Effect of recombinant superoxide dismutase (_r_SOD) supplementation on intestinal oxidative stress of growing pigs under thermoneutral (TN) or cyclic heat stress (_C_HS) conditions.

Variables ^1^	TN		_C_HS		SED ^2^	*p*-Value ^3^	
	CON	_r_SOD	CON	_r_SOD		T	D
Jejunum							
SOD, IU/mg	0.40	0.43	0.41	0.46	0.077	0.70	0.39
GP*_x_*, IU/mg	77.8	72.7	65.7	66.3	4.67	0.008	0.40
GSH:GSSG ^4^	0.345	0.318	0.316	0.285	0.0132	0.001	0.049
	(2.21)	(2.08)	(2.08)	(1.93)			
Ileum							
SOD, IU/mg	0.46	0.51	0.56	0.54	0.060	0.16	0.66
GP*_x_*, IU/mg	66.2	64.6	76.9	71.0	5.46	0.027	0.33
GSH:GSSG ^4,5^	0.505 ^a^	0.418 ^ab^	0.326 ^b^	0.434 ^ab^	0.0762	0.50	0.80
	(3.20)	(2.62)	(2.12)	(2.71)			

^1^ SOD: superoxide dismutase; GP*_x_*: glutathione peroxidase; GSH: reduced glutathione; GSSG: oxidised glutathione. ^2^ SED: standard error of the difference for the interaction between temperature and diet. ^3^ T: temperature; D: diet, T × D interactions were not significant for all parameters except where indicated. ^4^ log-transformed data and the back-transformed values were presented in parentheses. ^5^ T × D interaction (^a,b^ *p*-Value = 0.069). *n* = 16 pigs per group.

**Table 7:** Effect of recombinant superoxide dismutase (_r_SOD) supplementation on intestinal transepithelial resistance (TER) and permeability of growing pigs under thermoneutral (TN) or cyclic heat stress (_C_HS) conditions.

Variables ^1^	TN		_C_HS		SED ^2^	*p*-Value ^3^	
	CON	_r_SOD	CON	_r_SOD		T	D
TER, Ω.cm^2^							
Jejunum	38.4	36.6	35.1	33.2	4.13	0.31	0.63
Ileum	38.1	36.1	36.7	33.2	3.52	0.43	0.19
Colon	33.7	37.8	40.5	41.5	5.66	0.20	0.73
P_app_, 10^−4^ × cm/s							
Ileum	537	690	398	454	187	0.16	0.45
Colon	1190	1392	552	710	564	0.13	0.55

^1^ TER: transepithelial resistance_;_ P_app_: apparent permeability of 4 kDa fluorescein isothiocyanate-dextran. ^2^ SED standard error of the difference for the interaction between temperature and diet. ^3^ T: temperature; D: diet. T × D interactions were not significant for all parameters. *n* = 16 pigs per group.

## Data Availability

The data presented in this study are available on request from the corresponding author.

## References

[1] Huynh T.T., Aarnink A.J., Verstegen M.W., Gerrits W.J., Heetkamp M.J., Kemp B., Canh T.T. (2005). Effects of increasing temperatures on physiological changes in pigs at different relative humidities. J. Anim. Sci..

[2] White H.M., Richert B.T., Schinckel A.P., Burgess J.R., Donkin S.S., Latour M.A. (2008). Effects of temperature stress on growth performance and bacon quality in grow-finish pigs housed at two densities. J. Anim. Sci..

[3] Oliveira R.F., Moreira R.H.R., Abreu M.L.T., Gionbelli M.P., Teixeira A.O., Cantarelli V.S. (2018). Effects of air temperature on physiology and productive performance of pigs during growing and finishing phases. S. Afr. J. Anim. Sci..

[4] Hall D.M., Buettner G.R., Oberley L.W., Xu L., Matthes R.D., Gisolfi C.V. (2001). Mechanisms of circulatory and intestinal barrier dysfunction during whole body hyperthermia. Am. J. Physiol. Heart Circ. Physiol..

[5] Tabler T.W., Greene E.S., Orlowski S.K., Hiltz J.Z., Anthony N.B., Dridi S. (2020). Intestinal Barrier Integrity in Heat-Stressed Modern Broilers and Their Ancestor Wild Jungle Fowl. Front. Vet. Sci..

[6] Liu F., Cottrell J.J., Furness J.B., Rivera L.R., Kelly F.W., Wijesiriwardana U., Pustovit R.V., Fothergill L.J., Bravo D.M., Celi P. (2016). Selenium and vitamin E together improve intestinal epithelial barrier function and alleviate oxidative stress in heat-stressed pigs. Exp. Physiol..

[7] Chatterjee S., Dziubla T., Butterfield D.A. (2016). Chapter Two—Oxidative Stress, Inflammation, and Disease. Oxidative Stress and Biomaterials.

[8] Stuart L.M., Ezekowitz R.A. (2005). Phagocytosis: Elegant complexity. Immunity.

[9] Cottrell J.J., Le H.H., Artaiz O., Iqbal Y., Suleria H.A., Ali A., Celi P., Dunshea F.R. (2022). Recent advances in the use of phytochemicals to manage gastrointestinal oxidative stress in poultry and pigs. Anim. Prod. Sci..

[10] Ighodaro O.M., Akinloye O.A. (2018). First line defence antioxidants-superoxide dismutase (SOD), catalase (CAT) and glutathione peroxidase (GPX): Their fundamental role in the entire antioxidant defence grid. Alex. J. Med..

[11] Elgendey F., Al Wakeel R.A., Hemeda S.A., Elshwash A.M., Fadl S.E., Abdelazim A.M., Alhujaily M., Khalifa O.A. (2022). Selenium and/or vitamin E upregulate the antioxidant gene expression and parameters in broilers. BMC Vet. Res..

[12] Calik A., Emami N.K., Schyns G., White M.B., Walsh M.C., Romero L.F., Dalloul R.A. (2022). Influence of dietary vitamin E and selenium supplementation on broilers subjected to heat stress, Part II: Oxidative stress, immune response, gut integrity, and intestinal microbiota. Poult. Sci..

[13] Hori Y., Hoshino J., Yamazaki C., Sekiguchi T., Miyauchi S., Mizuno S., Horie K. (1997). Effect of lecithinized-superoxide dismutase on the rat colitis model induced by dextran sulfate sodium. Jpn. J. Pharmacol..

[14] Suzuki Y., Matsumoto T., Okamoto S., Hibi T. (2008). A lecithinized superoxide dismutase (PC-SOD) improves ulcerative colitis. Color. Dis..

[15] Lalles J.P., Lacan D., David J.C. (2011). A melon pulp concentrate rich in superoxide dismutase reduces stress proteins along the gastrointestinal tract of pigs. Nutrition.

[16] Ahasan A., Invernizzi G., Farina G., Pilotto A., Barbé F., Bontempo V., Rossi R., Bellagamba F., Lecchi C., Savoini G. (2019). The effects of superoxide dismutase-rich melon pulp concentrate on inflammation, antioxidant status and growth performance of challenged post-weaning piglets. Animal.

[17] Gasthuys E., Devreese M., Millecam J., Sys S., Vanderperren K., Delanghe J., Vande Walle J., Heyndrickx M., Croubels S. (2017). Postnatal Maturation of the Glomerular Filtration Rate in Conventional Growing Piglets as Potential Juvenile Animal Model for Preclinical Pharmaceutical Research. Front. Pharmacol..

[18] Wijffels G., Sullivan M.L., Stockwell S., Briscoe S., Anderson S.T., Li Y., de Melo Costa C.C., McCulloch R., Olm J.C.W., Cawdell-Smith J. (2023). Comparing the responses of grain fed feedlot cattle under moderate heat load and during subsequent recovery with those of feed restricted thermoneutral counterparts: Metabolic hormones. Int. J. Biometeorol..

[19] Cottrell J.J., Furness J.B., Wijesiriwardana U.A., Ringuet M., Liu F., DiGiacomo K., Leury B.J., Clarke I.J., Dunshea F.R. (2020). The Effect of Heat Stress on Respiratory Alkalosis and Insulin Sensitivity in Cinnamon Supplemented Pigs. Animals.

[20] Cui Y., Gu X. (2015). Proteomic changes of the porcine small intestine in response to chronic heat stress. J. Mol. Endocrinol..

[21] Le H.H., Shakeri M., Suleria H.A.R., Zhao W., McQuade R.M., Phillips D.J., Vidacs E., Furness J.B., Dunshea F.R., Artuso-Ponte V. (2020). Betaine and Isoquinoline Alkaloids Protect against Heat Stress and Colonic Permeability in Growing Pigs. Antioxidants.

[22] Xia B., Wu W., Fang W., Wen X., Xie J., Zhang H. (2022). Heat stress-induced mucosal barrier dysfunction is potentially associated with gut microbiota dysbiosis in pigs. Anim. Nutr..

[23] Igarashi R., Hoshino J., Ochiai A., Morizawa Y., Mizushima Y. (1994). Lecithinized superoxide dismutase enhances its pharmacologic potency by increasing its cell membrane affinity. J. Pharmacol. Exp. Ther..

[24] Carillon J., Barbé F., Barial S., Saby M., Sacy A., Rouanet J.M. (2016). Diet supplementation with a specific melon concentrate improves oviduct antioxidant defenses and egg characteristics in laying hens. Poult. Sci..

[25] Hou C.L., Zhang J., Liu X.T., Liu H., Zeng X.F., Qiao S.Y. (2014). Superoxide dismutase recombinant Lactobacillus fermentum ameliorates intestinal oxidative stress through inhibiting NF-κB activation in a trinitrobenzene sulphonic acid-induced colitis mouse model. J. Appl. Microbiol..

[26] Mamat U., Wilke K., Bramhill D., Schromm A.B., Lindner B., Kohl T.A., Corchero J.L., Villaverde A., Schaffer L., Head S.R. (2015). Detoxifying Escherichia coli for endotoxin-free production of recombinant proteins. Microb. Cell Factories.

[27] Wen X., Wu W., Fang W., Tang S., Xin H., Xie J., Zhang H. (2019). Effects of long-term heat exposure on cholesterol metabolism and immune responses in growing pigs. Livest. Sci..

[28] Victoria Sanz Fernandez M., Johnson J.S., Abuajamieh M., Stoakes S.K., Seibert J.T., Cox L., Kahl S., Elsasser T.H., Ross J.W., Isom S.C. (2015). Effects of heat stress on carbohydrate and lipid metabolism in growing pigs. Physiol. Rep..

[29] Lu Z., He X.F., Ma B.B., Zhang L., Li J.L., Jiang Y., Zhou G.H., Gao F. (2019). Increased fat synthesis and limited apolipoprotein B cause lipid accumulation in the liver of broiler chickens exposed to chronic heat stress. Poult. Sci..

[30] Qu H., Yan H., Lu H., Donkin S.S., Ajuwon K.M. (2016). Heat stress in pigs is accompanied by adipose tissue-specific responses that favor increased triglyceride storage. J. Anim. Sci..

[31] Perriotte-Olson C., Adi N., Manickam D.S., Westwood R.A., Desouza C.V., Natarajan G., Crook A., Kabanov A.V., Saraswathi V. (2016). Nanoformulated copper/zinc superoxide dismutase reduces adipose inflammation in obesity. Obesity.

[32] Natarajan G., Perriotte-Olson C., Casey C.A., Donohue T.M., Talmon G.A., Harris E.N., Kabanov A.V., Saraswathi V. (2019). Effect of nanoformulated copper/zinc superoxide dismutase on chronic ethanol-induced alterations in liver and adipose tissue. Alcohol.

[33] Ma M., Liu H., Yu J., He S., Li P., Ma C., Zhang H., Xu L., Ping F., Li W. (2020). Triglyceride is independently correlated with insulin resistance and islet beta cell function: A study in population with different glucose and lipid metabolism states. Lipids Health Dis..

[34] Grundy S.M. (1999). Hypertriglyceridemia, insulin resistance, and the metabolic syndrome. Am. J. Cardiol..

[35] McDermott B.P., Smith C.R., Butts C.L., Caldwell A.R., Lee E.C., Vingren J.L., Munoz C.X., Kunces L.J., Williamson K., Ganio M.S. (2018). Renal stress and kidney injury biomarkers in response to endurance cycling in the heat with and without ibuprofen. J. Sci. Med. Sport.

[36] Mansour S.G., Verma G., Pata R.W., Martin T.G., Perazella M.A., Parikh C.R. (2017). Kidney Injury and Repair Biomarkers in Marathon Runners. Am. J. Kidney Dis..

[37] Semenza J.C., McCullough J.E., Flanders W.D., McGeehin M.A., Lumpkin J.R. (1999). Excess hospital admissions during the July 1995 heat wave in Chicago. Am. J. Prev. Med..

[38] Hales J.R.S. (1973). Effects of exposure to hot environments on the regional distribution of blood flow and on cardiorespiratory function in sheep. Pflügers Archiv..

[39] Sils I.I., Szlyk-Modrow P.C., Tartarini K.A., Matthew C.B., Francesconi R.P. (2001). Effect of nitric oxide synthase inhibition on regional blood flow during hyperthermia. J. Therm. Biol..

[40] Shakeri M., Cottrell J.J., Wilkinson S., Zhao W., Le H.H., McQuade R., Furness J.B., Dunshea F.R. (2020). Dietary Betaine Improves Intestinal Barrier Function and Ameliorates the Impact of Heat Stress in Multiple Vital Organs as Measured by Evans Blue Dye in Broiler Chickens. Animals.

[41] Le H.H., Vidacs E., Phillips D.J., Zhao W., Furness J.B., Gabler N.K., Renaudeau D., Wijffels G., Dunshea F.R., DiGiacomo K. (2019). PSIV-8 Effect of selenium and superoxide dismutase supplementation on heat stressed pigs. J. Anim. Sci..

